# Acute Scrotum in Children: A Retrospective Study of Cases With Review of Literature

**DOI:** 10.7759/cureus.36259

**Published:** 2023-03-16

**Authors:** Roshan Chanchlani, Himanshu Acharya

**Affiliations:** 1 Pediatric Surgery, All India Institute of Medical Sciences (AIIMS) Bhopal, Bhopal, IND; 2 Department of Pediatric Surgery, Netaji Subhash Chandra Bose (NSCB) Medical College, Jabalpur, IND

**Keywords:** epididymorchitis, testis, testicular torsion, epididymitis, acute scrotum

## Abstract

Background: Testicular pain or swelling, often referred to as acute scrotum, can have a number of causes and presentations. Testicular torsion is an emergency condition requiring early diagnosis and surgery to salvage the involved testis in order to preserve testicular fertility. The study is aimed to know the incidence, aetiology, and management of acute scrotal conditions with a particular focus on testicular torsion. Epididymorchitis, trauma, and scrotal cellulitis are other causes of acute scrotum which are managed conservatively after proper investigations.

Material and methods: The authors retrospectively reviewed 10-year epidemiological data of all children age under 14 years admitted to the tertiary care hospital with the diagnosis of acute scrotum. Data were collected about the clinical history, physical examination, biochemical investigations, Doppler ultrasound, and management done.

Results: 133 children aged between 0 days and 14 years (mean age, 7.5 years) were found to have acute scrotum out of which 67 (50.37%) patients had epididymitis, 54 (40.60%) patients presented with Torsion testis, three (2.25%) had torsion of testicular appendages, eight (6.01%) had scrotal cellulitis and one (0.75%) presented with a strangulated hernia. Due to late presentation, testis could be salvaged in only eight of the 54 patients with torsion testis. The testicular loss was seen more in bigger children and those with signs of infection in blood reports and colour Doppler showing no blood flow in the testis.

Conclusion: The study results indicate that non-recognition of the severity of paediatric acute scrotum results in late presentation leading to testicular loss. Timely diagnosis will require sensitization of the parents, primary care providers, and paediatricians for this grave condition which leads to permanent testicular loss.

## Introduction

The acute scrotum is a common presentation in Paediatric Urology practice [1]. It is seen as early as the neonatal age group to the pubertal age. Out of all the conditions, testicular torsion remains an emergency, requiring early diagnosis and surgery to salvage the involved testis in order to preserve testicular fertility [2]. In 20% of patients presenting with epididymorchitis, some structural anomalies of the urinary tract may be associated. Strangulated inguinal hernia can also be one of the presentations of acute scrotum in children. Testicular torsion causes testicular injury mainly due to the rotation of the spermatic cord and its vessels leading to ischemia and oxidative damage of the testis [3].

On clinical examination, there is the presence of a swollen, erythematous, and tender scrotum. Duration of symptoms less than 24 hours, nausea or vomiting, a high position of the testis, and an absent cremasteric reflex is suggestive of testicular torsion. However, in newborns and infants, a strangulated inguinal hernia may present with acute scrotum. The patient outcome depends upon the time delay from the onset of pain to surgical intervention [4,5]. Testicular torsion is an emergency requiring a prompt diagnosis and surgery to avoid irreversible changes and a complete loss of the testis.

The surgical procedure implied is scrotal exploration followed by orchiectomy of the non-viable testis and bilateral orchidopexy of the contralateral testis. Trauma and infection are the other causes of acute scrotum requiring medical management. The present study is aimed to identify potential factors that may predict a testicular salvage in children presenting with acute scrotum.

## Materials and methods

This cross-sectional study was carried out in the Department of Paediatric Surgery in a tertiary care institute after written consent and clearance from the institutional review board with vide letter number "EC/New/Inst/2021/1682". The authors retrospectively reviewed 10-year epidemiological data of all children age under 14 years admitted to the tertiary care hospital with complaints of acute scrotum. The medical records of the patients were noted with respect to the clinical history, age, duration of symptoms, history of fever, vomiting, and trauma, examination findings were recorded including the side of the involved testis, erythema, swelling, scrotal tenderness, and fever. The findings of Doppler ultrasound, biochemical investigation, and surgical and non-surgical management were recorded. All boys presenting with acute scrotum were subjected to ultrasonography. However, immediate exploration was done in cases of suspected torsion of testis. Heterogeneous echogenicity was also presented in all cases of torsion who underwent orchidectomy. A prediction of the diagnosis was based on clinical and sonographic features and was followed by surgical exploration when needed. Postoperatively, these patients received analgesics. The patients were followed up after two weeks, three months, and then yearly. The patients with epididymoorchitis were followed for three years to look for episodes of urinary tract infection (UTI) and patients with torsion were followed to look for growth of the opposite testis. The data were tabulated in Microsoft Excel (Redmond, WA, USA) and presented as Numbers (Percentage).

## Results

In the present study, 133 children were enrolled and successfully completed the study. Out of total of 133 children, 17 were of age less than two years. However, most of the children were of age more than four years (66%) (Table 1). 

**Table 1:** Distribution of age

Age Groups	Number	Percentage
Less than or equal to 2 years	17	13.0%
2-4 years	28	21.0%
More than 4 years	88	66.0%

Table 2 depicts demographic and epidemiological profile of the patients. Out of total patients, 56.39% were from rural areas and 43.6% belonged to urban groups. On comparing education status, 88 and 45 were illiterates and literates respectively. The socioeconimic status of 91 (69.5%) was below the poverty line (Table 2). 

**Table 2:** Demographic and Epidemiological data of patients

Demographic Profile		Number	Percentage
Residential Area	Rural	75	56.39%
	Urban	58	43.6%
Education	Illiterate	88	66.15%
	literate	45	33.85%
Socio Economic Status	Above Poverty	42	31.5%
	Below Poverty	91	69.5%

The most common cause of acute scrotum in the present study was epididymorchitis in 67 (50.37%) of cases (Table 3). The mean age of presentation for epididymorchitis was 12 years of age in the study population. However, 54 (40.60%) patients had complaints of testicular torsion and 7.5 years was the mean age of presentation in such group (Table 3). Out of the total, one, three, and eight cases of incarcerated hernia, torsion of appendages of testis and scrotal cellulitis and infection respectively was observed. Incarcerated hernia and scrotal cellulitis and infection was observed in children of less than four years of mean age and torsion of appendages of testis was observed in patients of more than four years of age (Table 3). 

**Table 3:** Causes of Acute Scrotum

Condition	Total number of cases (percentage)	Mean age of presentation in years
Testicular Torsion	54 (40.60%)	7.5
Epididymorchitis	67 (50.37%)	12
Incarcerated hernia	01 (0.75%)	1
Torsion of appendages of testis	03 (2.25%)	11.5
Scrotal cellulitis and infection	08 (6.01%)	2.3

The most common presenting symptom of the patients was pain in 88 (66.16%) (Table 4). However, in 32 (24.06%) patients pain was associated with swelling. Redness and fever was observed in 10 (7.51%) and three (2.25%) of the cases (Table 4).

**Table 4:** Clinical presentation of patients

Presenting symptoms	Total number of cases	Percentage (%)
Pain	88	66.16
Pain and swelling	32	24.06
Erythema	10	7.51
Fever	3	2.25

Table 5 depicts the results of the management performed on the patients presented at our institute. Out of a total of 54 patients of testicular torsion, eight testes were salvaged. Scrotal cellulitis was seen in eight cases which were managed conservatively (Table 5). All cases of torsion of appendages of testis and one case of strangulated hernia were managed successfully by surgical exploration (Table 5).

**Table 5:** Results and Complication of different cases presented and treated

Type of cases	Number of Cases	Testis salvaged (Patients)
Testicular Torsion	54	8
Scrotal cellulitis	3	3
Torsion of appendages of testis	3	3
Strangulated Hernia	1	1
Total	61	

## Discussion

Acute scrotum in the pediatric population is a common presentation requiring early referral and prompt investigations for testicular salvage [1]. Pediatric testicular torsion is seen in infancy and then at 12 to 18 years of age [2,6]. Similar findings were noted in this series and in 66% of cases it was seen in the age groups of more than four years. Pain, swelling, erythema, and fever were common presentations of acute scrotum in this study. Most of the patients were illiterate and from poor socioeconomic status. Ghosh et al. in their study observed no pain in neonates presenting with acute scrotum as in one of our cases [3]. Laher et al. observed that there may be a history of trauma in such cases and the main diagnostic dilemma is to differentiate testicular torsion from epididymorchitis so that exploration of hemiscrotum can be planned [7].

In this series testicular torsion was seen in 40.60% of cases and epididymal-orchitis was found in 50.37% of cases. The high number of epididymo-orchitis may be due to the reason that most of the patients were of preadolescent age and had UTI. Patients with acute epididymitis had tender epididymis, while patients with torsion of the testis had tender testicles. Epididymitis in young children may not present with urinary tract infections. However, children and young adults presenting with any episode of epididymitis and urinary tract infection should be investigated with Ultrasonography (USG) of the kidneys and bladder. If any structural defect is identified a voiding cystourethrogram should be done. Treatment includes antibiotic therapy, analgesics, rest, scrotal support, and elevation. Sometimes in a patient presenting with a history of trauma the diagnosis of testicular torsion cannot be ruled out. Pain persisting for more than one to two hours after trauma is not normal and calls for investigations to rule out testicular torsion. On-and-off testicular pain suggests intermittent torsion with spontaneous torsion de-torsion syndrome. Delayed diagnosis of testicular torsion leads to testicular loss for the patient and also medicolegal consequences for the treating surgeon [8]. Therefore, when the patients present early there should not be any further delays in operating the patients.

**Figure 1:**
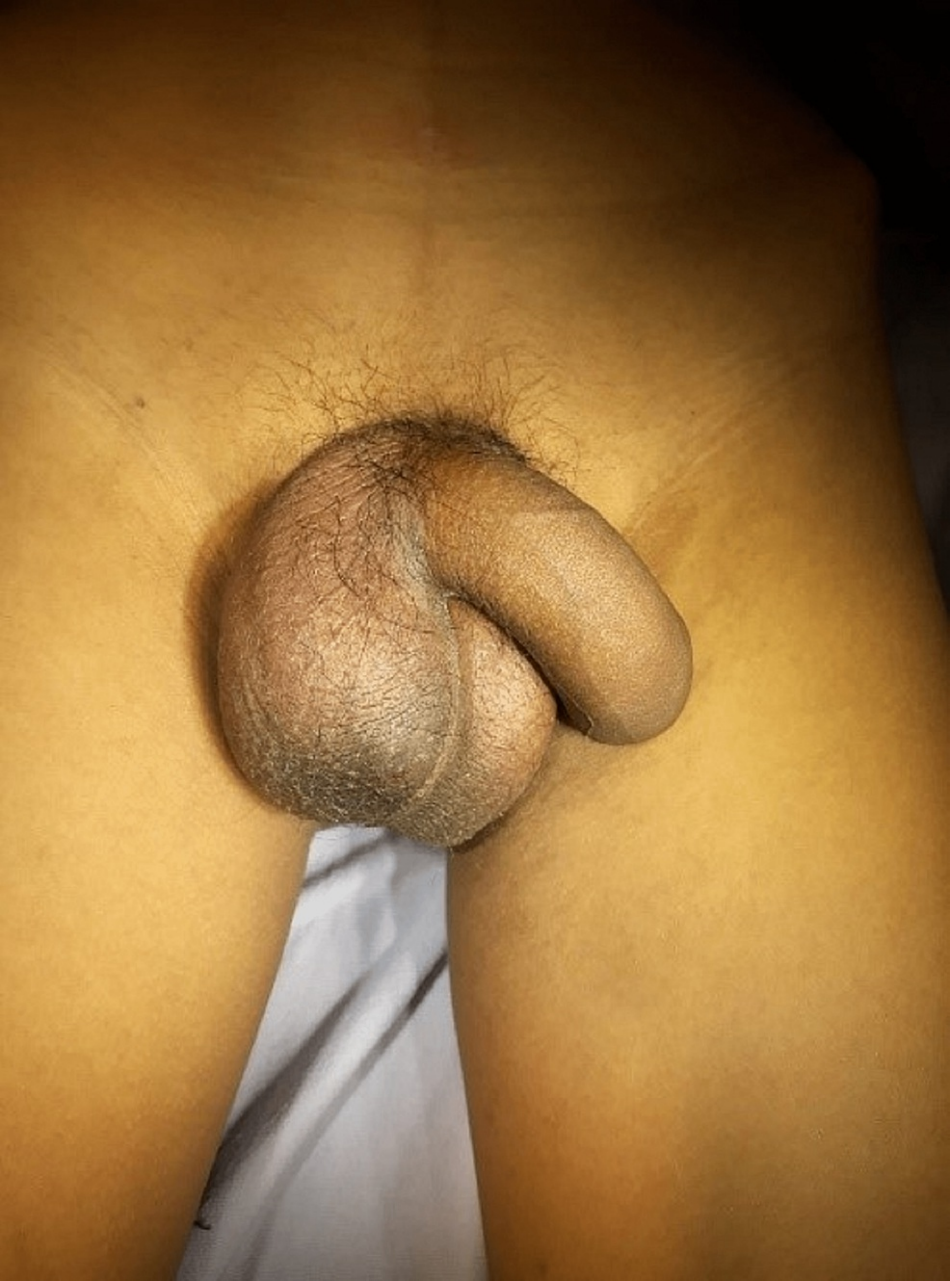
Clinical presentation of torsion of right testis

**Figure 2:**
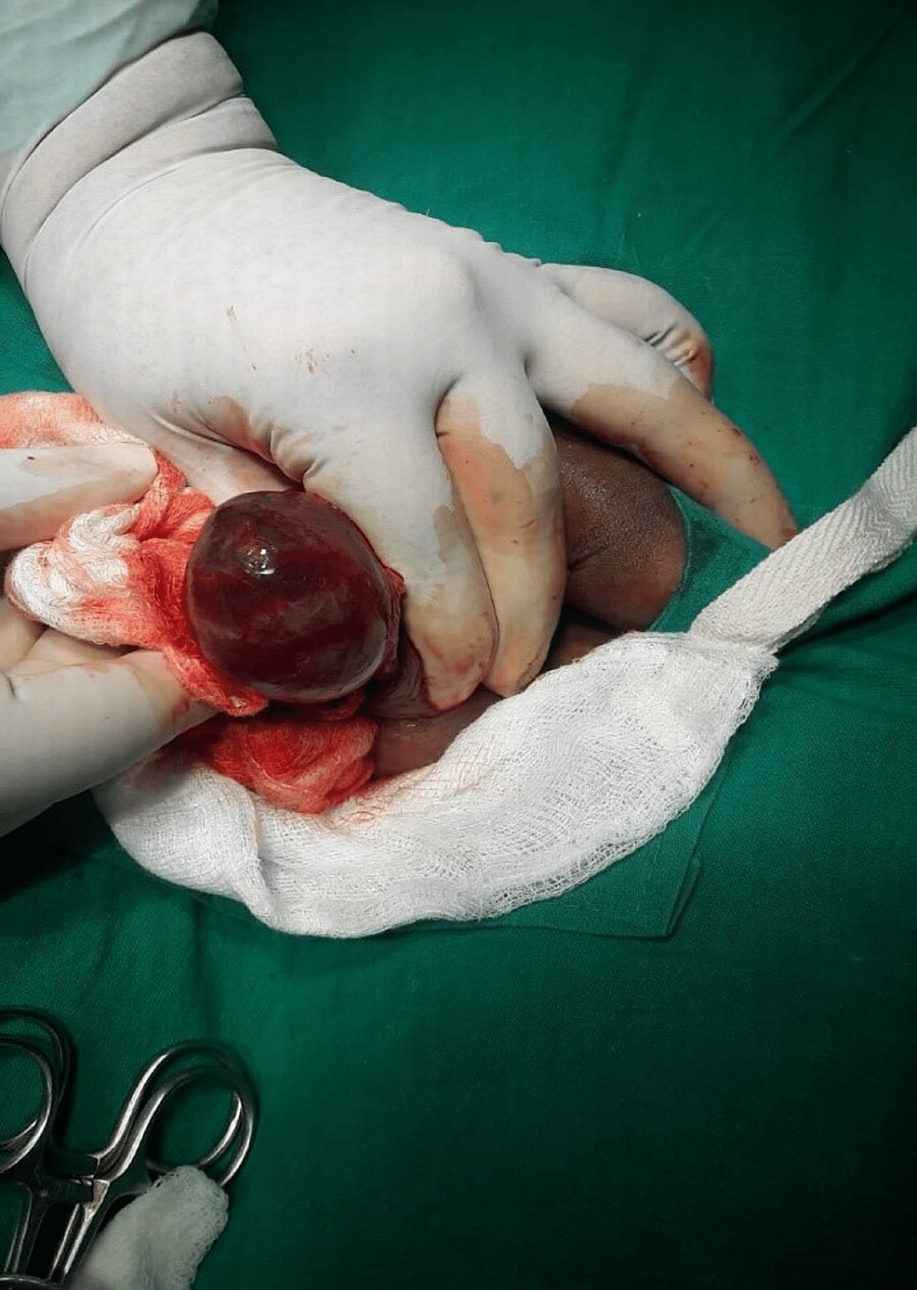
Intraoperative presentation of gangrenous right testis

Dupond et al. in a multicentric study observed testicular ischemia and risk of testicular loss in 72% of patients if they were referred more than six hours after the onset of symptoms [9]. According to Schalamon et al., in about 16% of cases, Doppler ultrasound remained unclear regarding the diagnosis of acute scrotum [10]. There is no specific investigation modality that can surely differentiate testicular torsion and epididymorchitis [10]. In a series of studies conducted by authors, it was observed that by doing color doppler in acute scrotal conditions, there was an improvement in the diagnosis, leading to a lesser need for scrotal exploration [11-13]. But, as it is operator dependent, sometimes an increased flow may be seen in testicular torsion [11-13] (Figure 1, 2). Yadav et al. suggested that trans scrotal Near Infrared Spectroscopy is a newer diagnostic test that holds promise for the future and a focused training program for pediatric urologists and radiologists will help to improve the quality of care in these patients [14].

A newer modality to differentiate epididymorchitis from testicular torsion is being proposed like mean platelet volume (MPV) [15]. It is shown that MPV is increased in cases with testicular torsion and therefore it could be the indicator of testicular viability [15]. Several authors in their study have highlighted that despite of improved diagnostic facilities in case of confusion prompt scrotal exploration in the patient is warranted [16-19]. Chun et al. in their study found the late presentation of acute scrotum leads to testicular loss. There are various reasons including less awareness among parents, caregivers, and treating physicians regarding the severity of the condition [20]. Pre-adolescent patients sometime present late due to shyness regarding conveying their condition to parents. The protective effect of cerium oxide (CeO_2_) on testicular function is being explored in experimental studies [21]. CeO_2_ reduces oxidative stress, cell trauma, and death in testis tissue in experimental rats. In this study rats in the CeO2 group demonstrated significantly milder tissue lesions compared with those in the control group [21]. 

The role of fixation of the contralateral testis is also being studied and practiced at different centers [22-24]. Prognosis is good when detorsion of the affected testis is performed within the first six hours. Duration of symptoms is the only predictor of successful testicular salvage following testicular torsion in children. It is associated with a substantial risk of testicular loss and atrophy [25].

Limitations of the study are that a standard protocol for the management of acute scrotum is required for avoiding diagnostic errors in these patients; also, we didn't evaluate the degree of torsion, like 180 or 360, or 720 degrees of torsion in the prediction of the success of testicular torsion.

## Conclusions

A detailed history, clinical examination, and diagnostic evaluation are most important in patients presenting with acute scrotum. However, emergency scrotal exploration is advocated in patients with suspected cases of torsion testis. Doppler Ultrasonography is a noninvasive method, for confirmation of the diagnosis. The study results indicate that non-recognition of the severity of paediatric acute scrotum results in late presentation leading to testicular loss. These patients must be evaluated and managed urgently and whenever a torsion is suspected Pediatric surgeons should be prompt for surgical testicular exploration.
